# Experimental study on the relationship between the mineral production capability and the physiochemical properties in the coproduction of Q phase-3CaO·3Al_2_O_3_·CaSO_4_ cement clinker

**DOI:** 10.1371/journal.pone.0195505

**Published:** 2018-04-10

**Authors:** Chao He, Chaochao Tian, Gang Li, Yahe Mei, Quanguo Zhang, Youzhou Jiao

**Affiliations:** 1 Key Laboratory of New Materials and Facilities for Rural Renewable Energy, Ministry of Agriculture, College of Mechanical & Electrical engineering, Henan Agricultural University, Zhengzhou, China; 2 Collaborative Innovation Center of Biomass Energy, Zhengzhou, Henan Province, China; Aligarh Muslim University, INDIA

## Abstract

A coproduction tests of quaternary (Q) phase(6CaO·4Al2O3·MgO·SiO2) -3CaO·3Al_2_O_3_·CaSO_4_ cement clinker and an experimental study on the relationship between the mineral production capability and the physiochemical properties are conducted in a two-stage multiphase reaction test bed with Changguang coal. X-ray diffractometer (XRD) analyses are performed on the coproduction clinker samples. The results demonstrate that, with the reduction in particle sizes of the coal powder and the additives and expanded screening level differences between them, both the proportion of Q phase and the mass of 3CaO·3Al_2_O_3_·CaSO_4_ in the clinker increase accordingly. When mixed coal powder particles are prepared through reducing particle sizes and expanding screening level differences between coal powder and additives, the additives CaO and MgO are more likely to be enclosed by coal powder to form globular polymerized particles. In addition, this preparation aids in polymerization and promotes even distribution of CaO, MgO and coal minerals, thus facilitating clinker mineral formation reactions of inorganic substances in the mixed coal powder. Target minerals, such as 2CaO·SiO_2_ and Q phase, are found in both industrial high-calcium limestone and low-calcium limestone coproduction clinker samples. A diffraction peak of free CaO is also evident in both samples. Compared with a coproduction clinker sample of high-calcium limestone, that of low-calcium limestone exhibits higher diffraction peaks for 2CaO·SiO_2_ and Q phase. With the current state of the art, it is not yet the optimum choice to substitute CaCO_3_ for CaO in Q-phase cement clinker coproduction. Before the technology matures and gains practical application, further study on the form and the mixing process of calcium-based additives for cement clinker coproduction will be required.

## 1. Introduction

**Fig 1:**
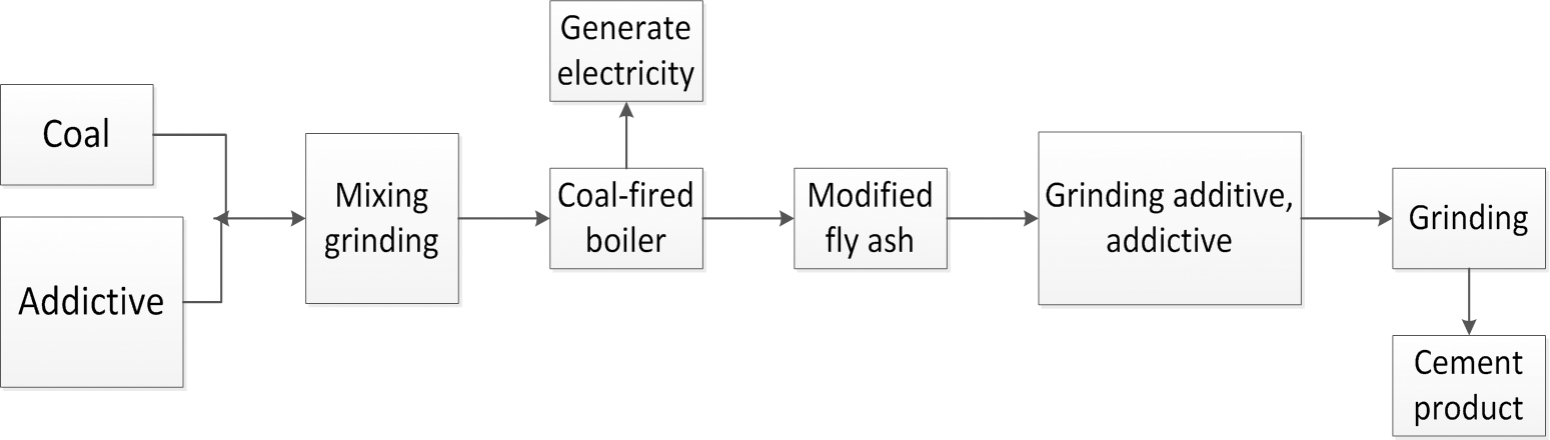
Diagram of the coproduction process of cement clinker in a power plant pulverized coal boiler.

Researchers have long been seeking methods to leverage the abundant mineral contents of silicon and aluminum in coal. One goal is to mix the proper quantities of calcium-based additives during the coal blending process for electric power generation as a function of the cement clinker ratio, to adjust the fly ash mineral composition during mixed coal powder combustion, desulfurization and ashing in a boiler and achieve the coproduction of fly ash with properties similar to cement clinker. Additionally, coal-fired power and cement industries are integrated into a high level of recycling as part of their economy [1–9]. Fig 1 shows a diagram of the coproduction process of cement clinker in a power plant pulverized coal boiler.

Kirgiz, M.S. performed an experimental on fresh and hardened properties of green binder concrete containing marble powder and brick powder. Results reveal that since the nanographite particle and the super plasticizer are added for mortar containing 35% MP and 65% pure cement, the mortar achieves greater flexural strength gain and compressive strength gain than over 45 and 5.2 (MPa) respectively[10]. O.M. Sharonova *et al* conducted a study on the chemical composition, mineral composition and hydration property of high-calcium fly ash collected by the electric precipitator in a power plant. They used a CaO-SiO_2_-Al_2_O_3_ phase diagram to analyze all possible mineral formation reactions of the high-calcium fly ash [6]. Luo Yuping *et al* performed mineral composition X-ray diffraction (XRD) analyses and cement physical property tests on the combustion residue of the mixed coal powder. They also studied the thermal effect of mixed coal powder. Their results showed that the optimum mixture ratio is 15% of calcium carbonate in the test coal powder and the coal powder combustion residue can be used as ordinary 32.5-grade Portland cement under this mixture ratio[2]. Wang Wenlong *et al* performed an experimental and theoretical study on the direct coproduction of high-silica sulfoaluminate cement clinker in a pulverized coal boiler. Their results showed that when the temperature is of 1300°C and the material remains for 7s in the high-temperature area, suspension combustion and simultaneous sintering of the cement clinker with the minerals 2CaO·SiO_2_ and 3Ca O·3Al_2_O_3_·CaSO_4_ are achieved. The mineral composition of coproduced high-silica sulfoaluminate cement clinker includes 2CaO·SiO_2_ and 3CaO·3Al_2_O_3_·CaSO_4_, and approximately 25% inactive compounds such as 2CaO·Al_2_O_3_·SiO_2_[3,4,11,12]. Liu Hao *et al* conducted a study on the migration and the multiphase reaction of Ca minerals in coal combustion solid residue. Their results showed that the Ca minerals in the coal combustion solid residue participate primarily in the sulfur fixation reaction and the solid phase reaction. When the temperature range supports the stable existence of CaSO_4_, the sulfur fixation reaction is more likely to occur than the solid phase reaction. As the temperature increases, the sulfur fixation product of CaSO_4_, decomposes or reacts with the oxides in the ash to produce more complex sulfur minerals, e.g., C_4_A_3_S and 2C_2_S·CaSO_4_, which causes the migration of Ca toward the silicate minerals. The primary Ca minerals produced by the solid phase reaction of calcium-enriched coal are CS, C_2_S, C_3_S, C_3_S_2_, C_2_F, CA, C_3_A, CAS_2_, C_2_AS, C_4_AF, C_3_FS_3_ and C_3_AS_3_. With the increase of CaO content, the quantity of high alkaline minerals in the ash gradually increases. The primary minerals produced in the ash (ordered by largest quantity produced) migrate in the order of A_3_S_2_-CAS_2_-C_2_AS-C_2_S. The formation of calcium-rich crystal minerals and vitreous minerals in the calcium-enriched solid ash slag enhances the hydration property of the ash slag [13]. Zhao Yongchun *et al* performed a study on the composition and the evolution mechanism of high-calcium coal ash. A systematic analysis of the lignite low-temperature mineral phase composition and high-calcium ash chemical composition led them to conclude that high-calcium fly ash particles have significantly diversified chemical compositions, based on the element type and content distribution in an individual particle, there are four primary categories, i.e., the calcium oxide phase, the calcium sulfate phase, the calcium aluminosilicate phase and the *Ca-S-X* phase, where *X* = *Fe*, *Al*, *Si*, *Mg*, *etc*.) [14].

In the early stage of this study, our research team performs comparative analyses on the raw material characteristics, the sintering conditions, the chemical composition, the mineral composition and the hydration properties of various types of cements and clinkers. Comparative analyses are also conducted on the chemical composition characteristics of coal minerals and coal ash contents versus cement silicon and aluminum raw materials, such as clay and bauxite. Based on a theoretical feasibility analysis of the coproduction of conventional cement clinkers in a pulverized coal boiler, such as silicate, aluminate and sulfoaluminate, a Q-phase mineral with low basicity, low sintering temperature and excellent hydration is introduced into the same technology, to coproduce a Q-phase cement clinker in a pulverized coal boiler. The concepts of the coproduction of Q-phase cement clinker by theoretical and experimental in the pulverized coal boiler are also proposed. The systematic experimental study is performed in a two-stage multiphase reaction test bed, and the theoretical study is conducted on the mineral formation mechanism of the coproduction of Q-phase cement clinker in the pulverized coal boiler [15–16].

However, further improvements in the clinker mineral formation capability of mixed coal powder particles and breakthroughs in theory and technology for cement clinker coproduction require additional theoretical and experimental study of the coal powder and additive particle gradation, the bonding mechanisms of the mixed coal powder particle and the additives, the control and optimization of the bonding method and the states of the coal powder and the additives in the coal powder particle mixture, and the adjustment and improvement of the coal powder particle unit reaction capability. During this early-stage experimental study on cement clinker coproduction in a pulverized coal boiler, the additives are primarily the analytical reagents of CaO and MgO. However, in common industrial practice, it is impractical to use chemical analytical reagent of CaO and MgO as additives. Hence, this study is conducted on the relationship between the physiochemical bases and the unit clinker mineral production capability of the material particles, such as the mixed coal powder preparation method, the forms of calcium-based additives and so on.

## 2. Test description

**Fig 2:**
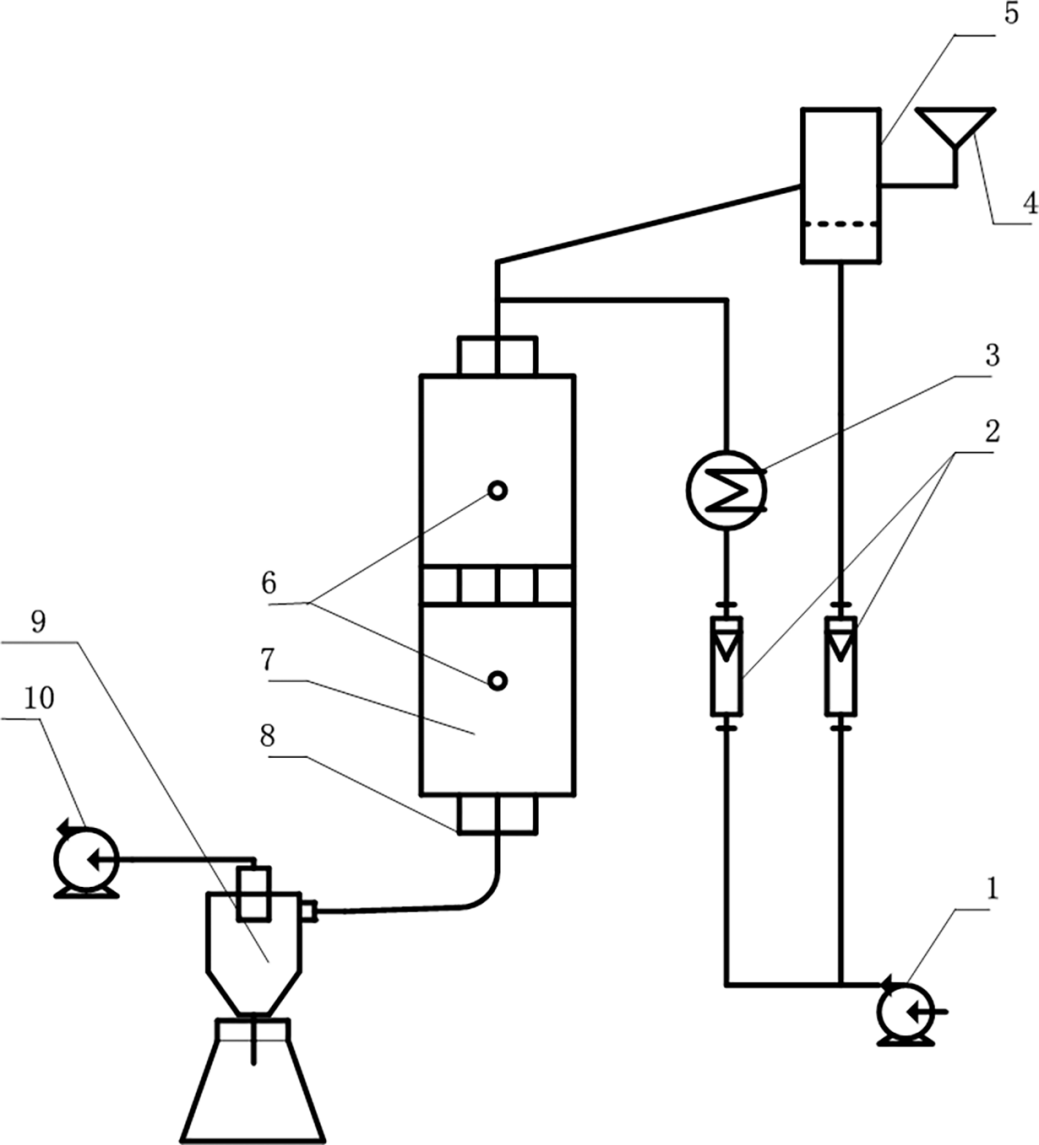
Diagram of two-stage multiphase reaction test bed. 1: Air compressor2: Rotor flow meter 3: Air preheater 4: Gunk 5: Fluidized bed feeder 6:Thermocouple7: Furnace body 8:Water-cooled jacket 9: Cyclone dust collector 10:Induced draft fan.

The experimental study on the relationship between the unit clinker mineral production capability of the material particles and the physiochemical base is conducted in a two-stage multiphase reaction test bed. Fig 2 shows a diagram of the test bed components. Laboratory bench mainly include, the feeding part, furnace body, the ash collecting part, air supply system and control system. During the test, the boiler is heated to a predefined temperature. Then, mixed coal powder is placed in the fluidized bed feeder, and the air compressor and induced draft fan are turned on to adjust the air flow. When the air passes the feeder, it carries mixed coal powder and injects it into the boiler. The mixed coal powder completes the combustion, sulfur fixation and ashing processes in the boiler. The coproduced clinker is immediately chilled with a water-cooling system and then collected using a vortex ash collector. The feeding quantity for the reaction test bench is of 3.5g/min with the air flow rate at 1m^3^/h and the residence time of6.94s for the materials by increasing the proportion of primary air to adjust the atmosphere in the furnace.

The high ash and grey Changguang coal were used for the experiment, andthe properties and the ash content analysis of the test coal are shown in Table 1 and Table 2, respectively. Coal elemental analysis and industrial analysis were carried out by means of the CHNS-932 elemental analyzer, purchased from the LECO laboratories in the United states. And the composition analysis of coal ash was carried out by using XJK12 chemical analyzer. CaO and MgO adopted in the experiment is chemical pure. The content analysis of industrial high calcium limestone and low calcium limestone are shown in Table 3.The pulverized coal after grinding the ball mill will be screened through 250 mesh square holes, and the particle size of pulverized coal should be controlled below 61μm. Meanwhile CaO and MgO were screened by 300 mesh square sieve respectively, and the particle size was controlled below 49μm. The mixed coal powder is prepared according to the batching scheme given in Table 4, and then the prepared mixed coal powder will be pulverized together to make the mixture well mixed. The preparation method of the mixed pulverized coal is marked as M1 to M6. The setting of the test table is shown in Table 5. Grind the mixed coal powder together to make it mixed uniformity and mark the mixed coal powder separably, and number the clinker calcined samples. The mineral composition of the collected clinker sample is then analyzed with XRD. The X ray diffraction analysis of mineral composition was carried out by using Japanese physics, Rigaku B/Max IIIB, and automatic X ray diffractometer. And the samples were analyzed by scanning electron microscope, using s-600 spectrometer supplied by Cambridge, England. The mineral composition, XRD and SEM analysis of the samples were completed at the analysis and test center of Zhejiang University,P.R.China.

**Table 1:** The properties analysis of the test coal.

Proximate analysis W_B_/%				Q_net,ad_ /(kJ·kg^-1^)	Elemental analysis W_B_/%				
*M*_ad_	*A*_ad_	*V*_ad_	FC_ad_		C_ad_	H_ad_	N_ad_	S_ad_	O_ad_
2.04	46.97	25.65	25.34	16 909	40.80	2.96	1.01	4.60	2.16

W_B_-Quality fraction, Q_net,ad_-The net calorific power of air-dried basis, *M*_ad_-The moisture of air-dried basis, *A*_ad_-The ash of air-dried basis, *V*_ad_-The volatilization of air-dried basis, FC_ad_-The fixed carbon of air-dried basis, C_ad_-The carbon content of air-dried basis, H_ad_-The hydrogen content of air-dried basis, N_ad_- The nitrogen content of air-dried basis, S_ad_- The sulfur content of air-dried basis, O_ad_- The oxygen content of air-dried basis. The noted has been added in the paper.

**Table 2:** The ash content analysis of the test coal %.

*w*(SiO_2_)	*w*(Al_2_O_3_)	*w*(CaO)	*w*(Fe_2_O_3_)	*w*(MgO)	*w*(K_2_O)	*w*(Na_2_O)
53.21	26.30	9.23	5.12	1.07	0.32	0.49

**Table 3:** The content analysis of industriallimestone*%*.

The type of limestone	*w*(Loss)	*w* (SiO_2_)	*w*(Al_2_O_3_)	*w*(Fe_2_O_3_)	*w*(CaO)	*w*(MgO)	∑
Low calcium	38.62	9.16	1.77	0.73	44.56	3.69	98.53
High calcium	39.63	6.75	0.75	0.23	51.1	0.35	98.81

**Table 4:** The preparation methods of mixed pulverized coal.

Serial number	Sieve size of pulverized coal(μm)	Sieve size of CaO (μm)	Sieve size of CaCO_3_ (μm)	Sieve size of MgO (μm)
M_1_	49	49	-	49
M_2_	61	49	-	49
M_3_	61	61	-	61
M_4_	80	61		61
M_5_	80	80	-	80
M_6_	61	-	49	49
M_7_	61	-	49	49

**Table 5:** The proportioning options and parameter setting of the test bed.

Serial number	*w*(pulverized coal) /%	*w* (CaO) /%	*w*(CaCO_3_) /%	*w* (MgO) /%	θ /°C	q_V,air_ /(m^3^.h^-1^)	Retention time /s	Input /(g.min^-1^)	Furnace atmosphere
M_1_	62.5	35	0	2.5	1 330	1	6.94	3.5	oxidation
M_2_	62.5	35	0	2.5	1 330	1	6.94	3.5	oxidation
M_3_	62.5	35	0	2.5	1 330	1	6.94	3.5	oxidation
M_4_	62.5	35	0	2.5	1 330	1	6.94	3.5	oxidation
M_5_	62.5	35	0	2.5	1 330	1	6.94	3.5	oxidation
M_6_	57	0	42	1	1 330	1	6.94	3.5	oxidation
M_7_	53.5	0	45	1.5	1 330	1	6.94	3.5	oxidation

## 3. Test results

Figs 3–9 depict XRD spectra for the major mineral components of the clinker samples obtained from calcination. Figs 10 and 11 show the SEM images for mixed coal powder samples M_2_ and M_3_, respectively. A diagram about mineral composition has been added to the supplement, for energy spectrum analysis, and which isshown in Figs 12 and 13.

**Fig 3:**
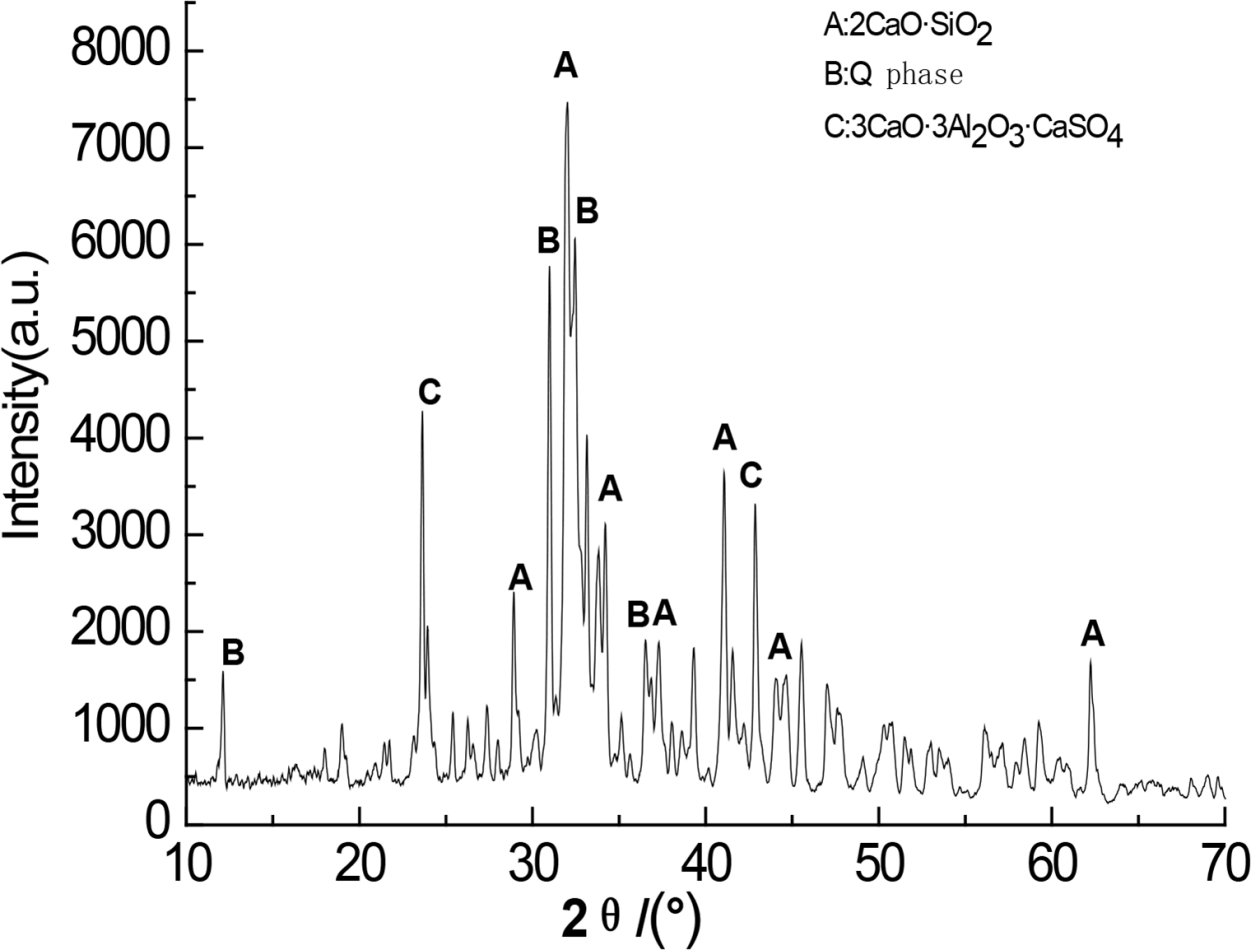
XRDspectra for the major mineralcomponents of the clinker sample M1.

**Fig 4:**
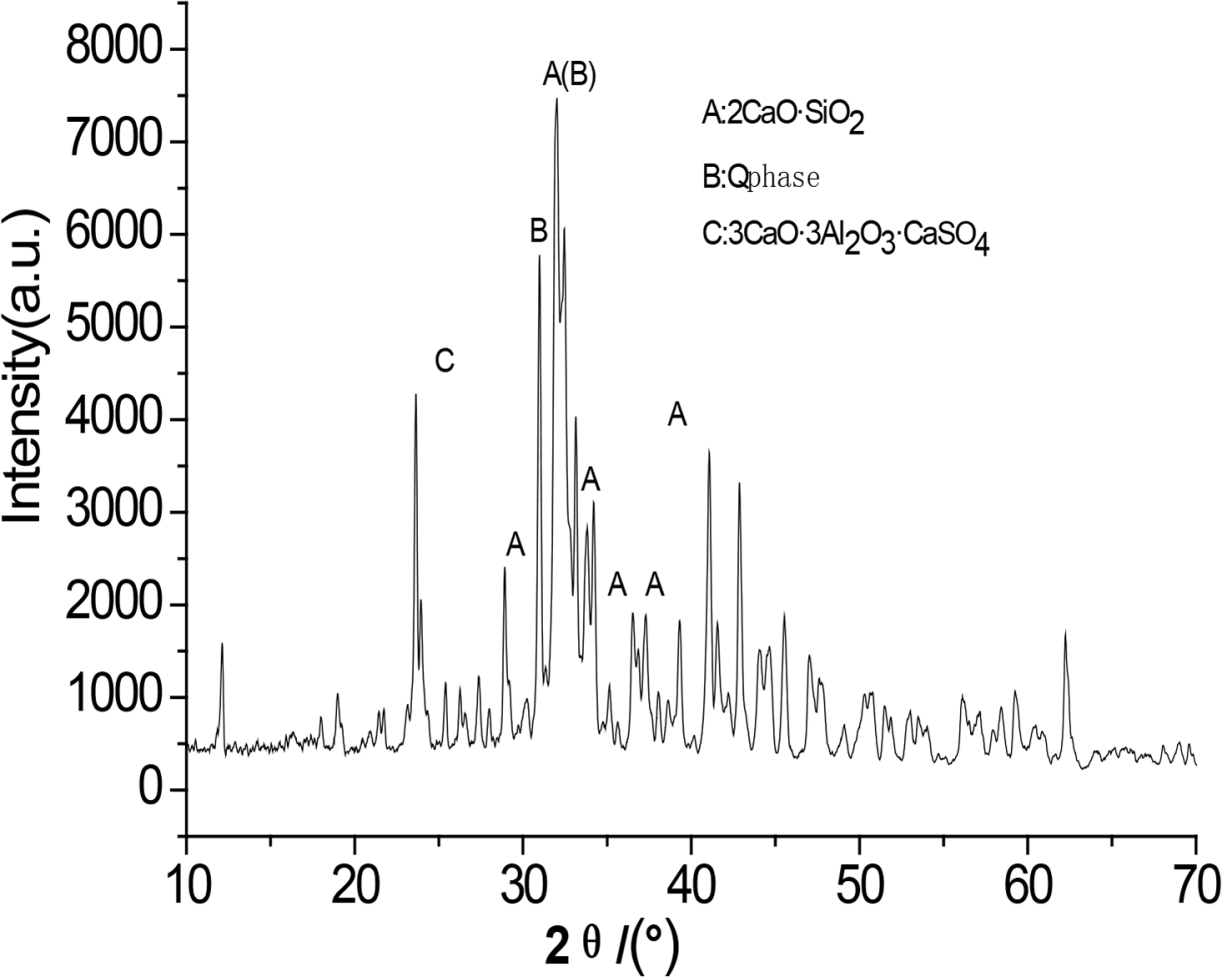
XRD spectra for the major components of the clinker sample M2.

**Fig 5:**
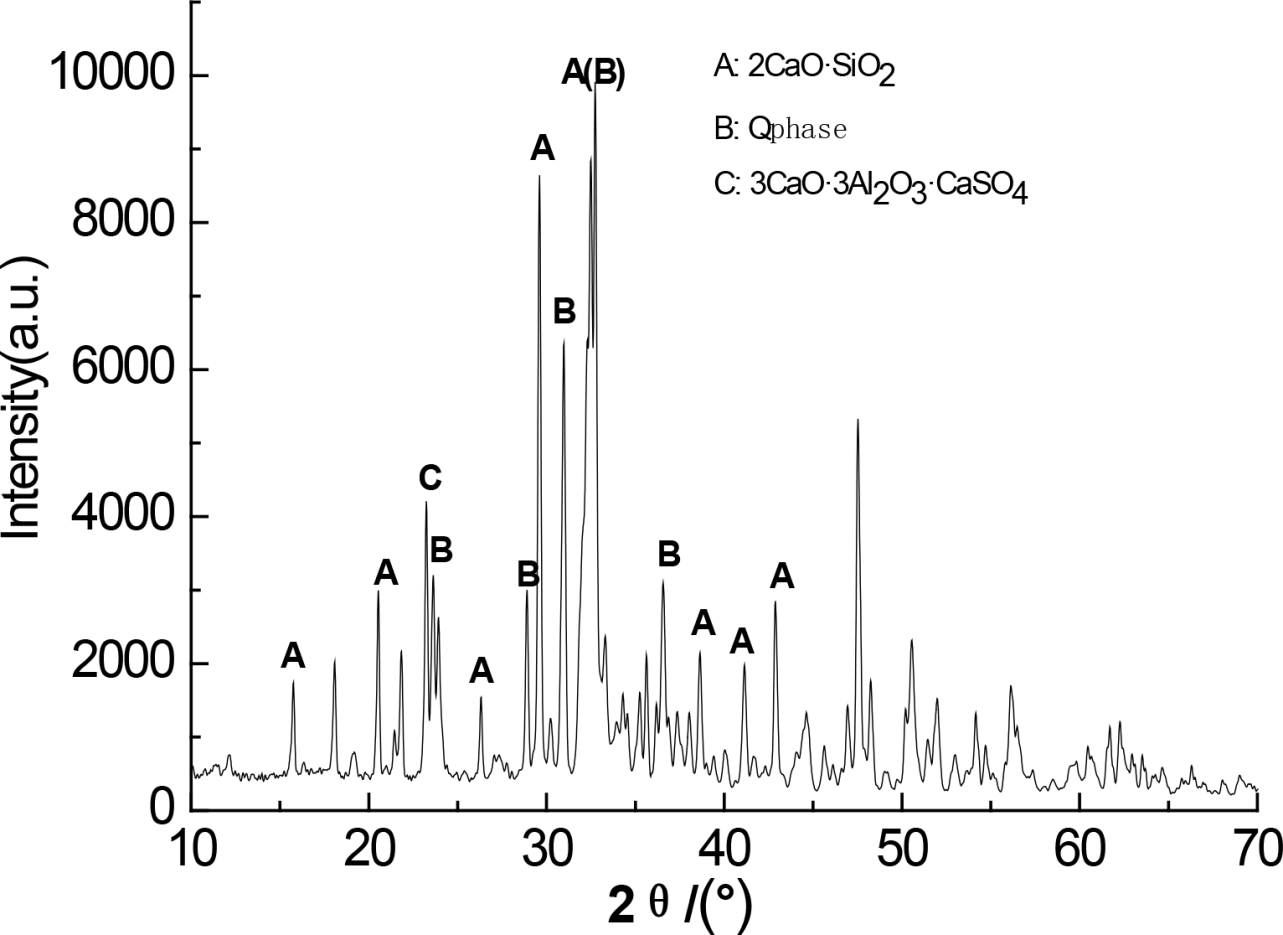
XRD spectra for the major mineral components of the clinker sample M3.

**Fig 6:**
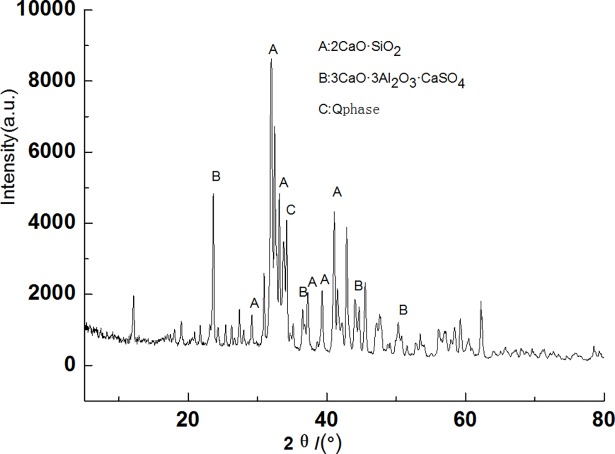
XRD spectra for the major mineral components of the clinker sample M4.

**Fig 7:**
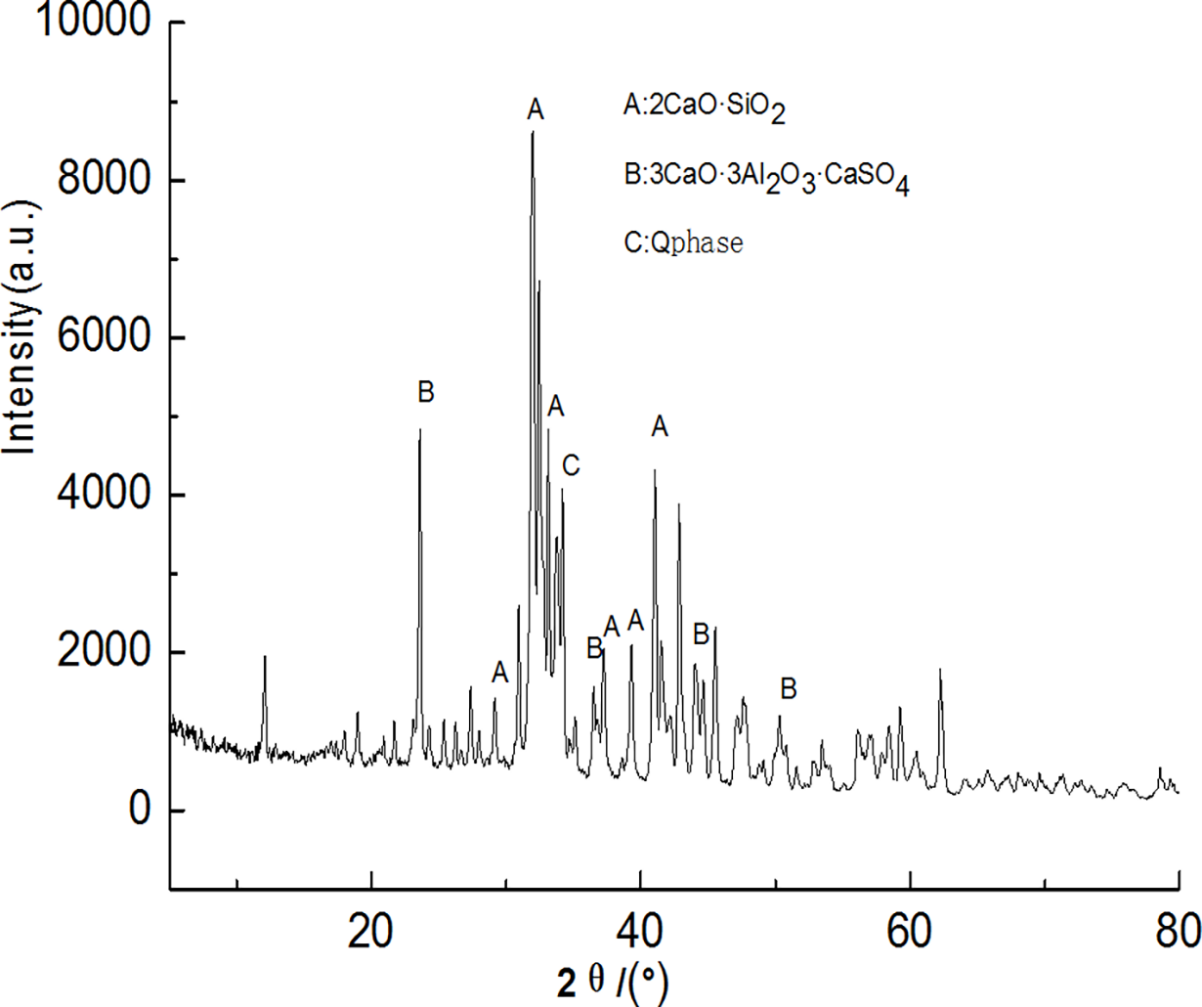
XRD spectra for the major mineral components of the clinker sample M5.

**Fig 8:**
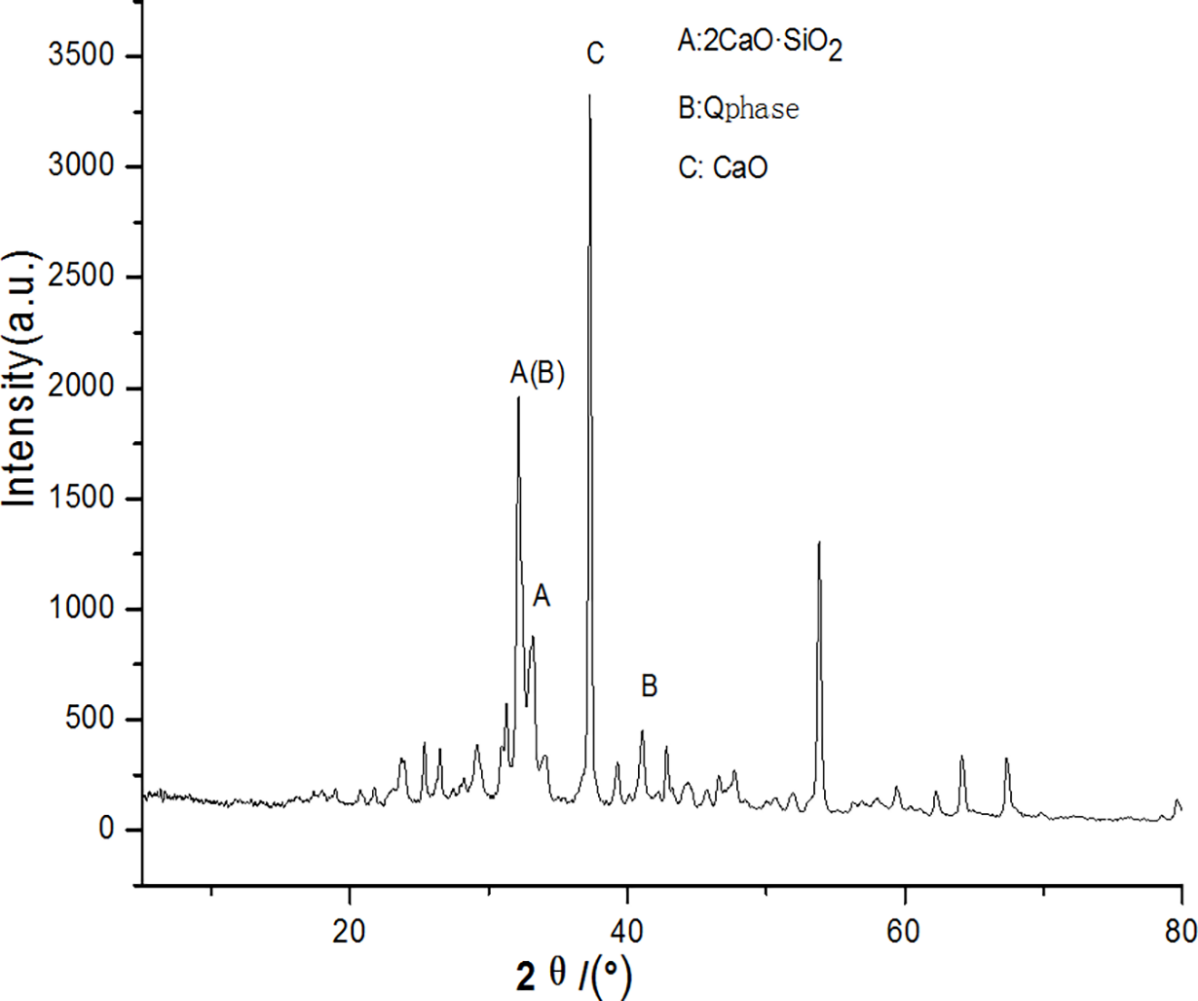
XRD spectra for the major mineral components of the clinker sample M6.

**Fig 9:**
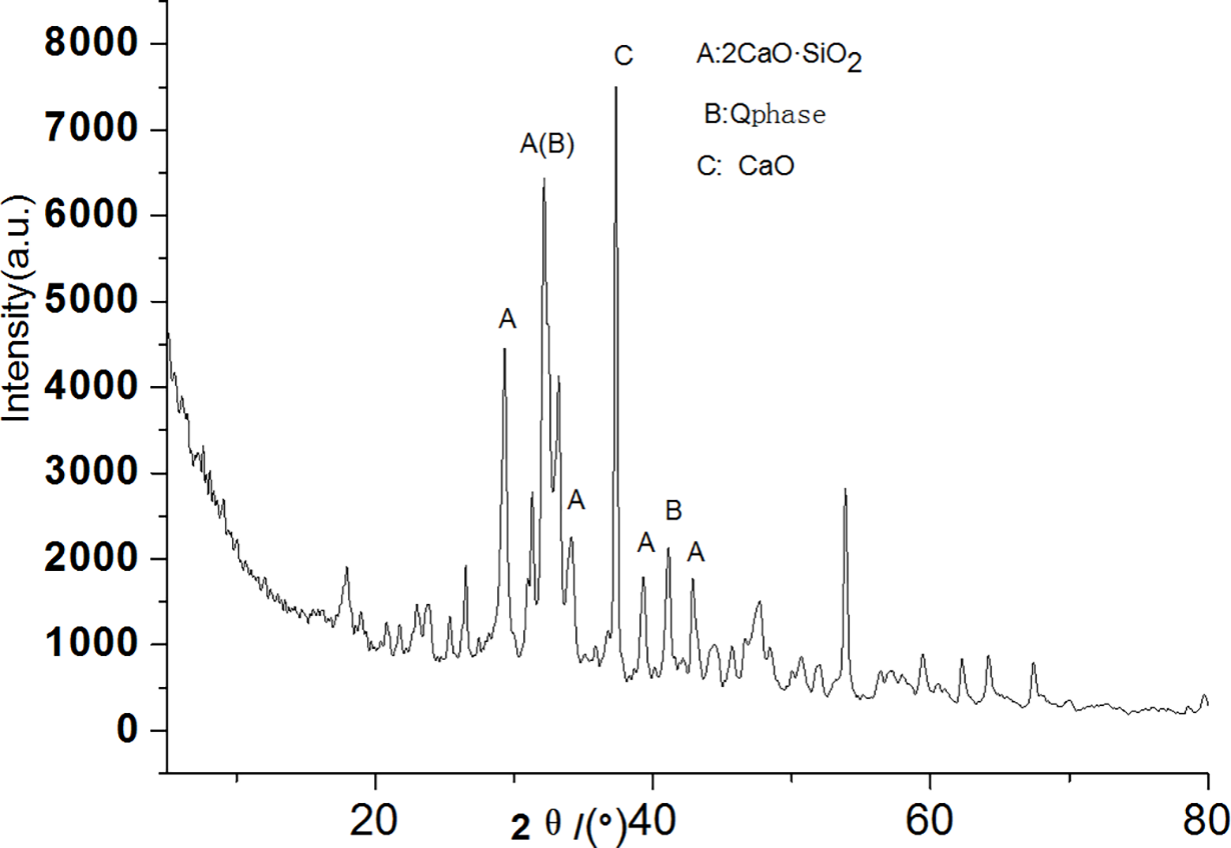
XRD spectra for the major mineral components of the clinker sample M7.

**Fig 10:**
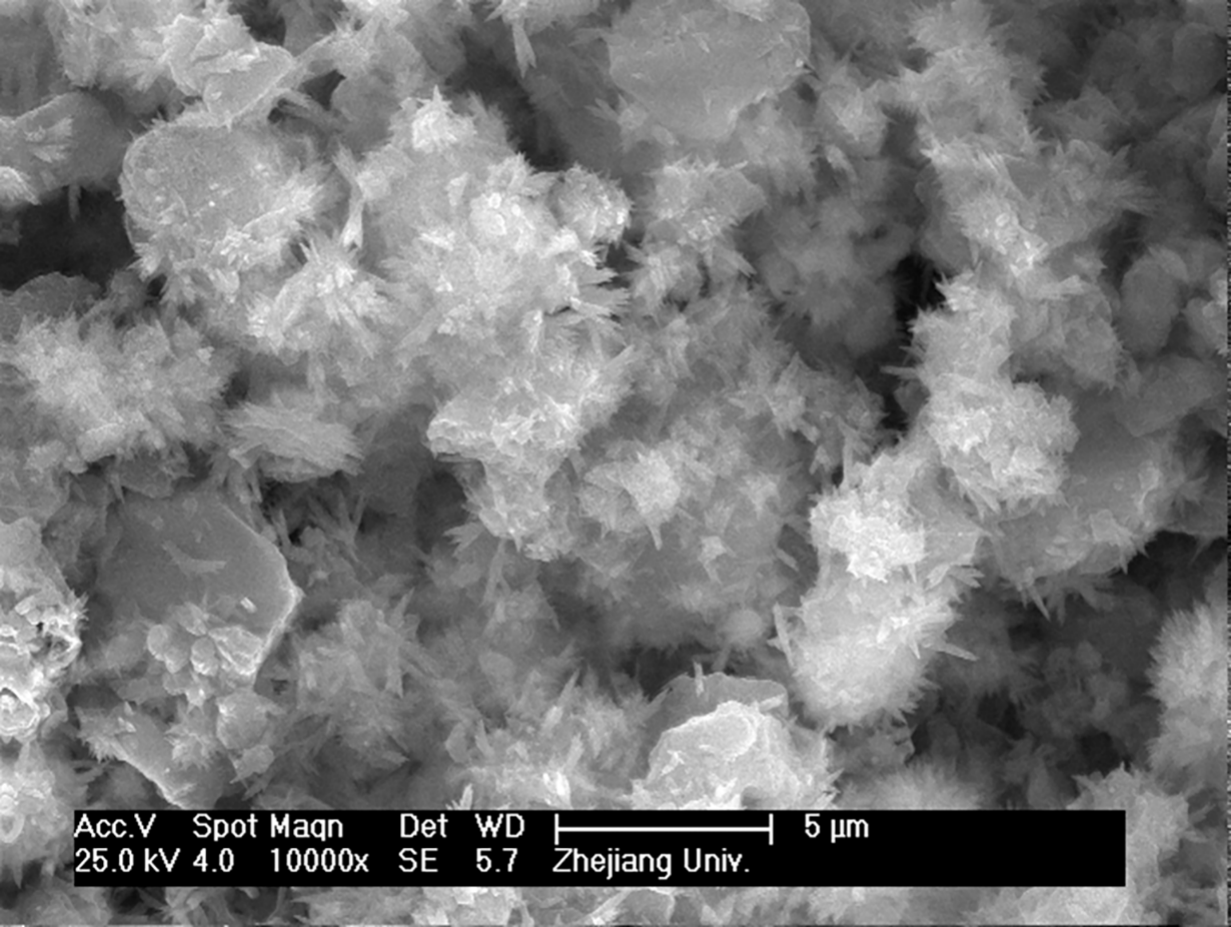
The SEM micrograph of globular mixed pulverized coal M2.

**Fig 11:**
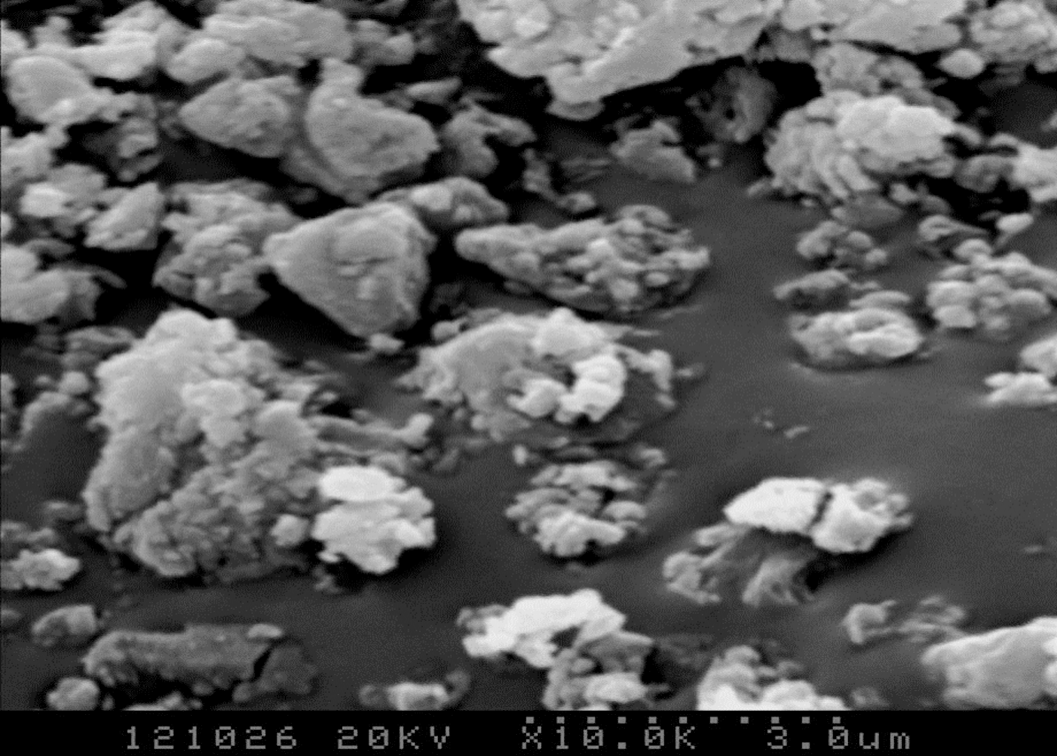
The SEM micrograph of globular mixed pulverized coal M3.

**Fig 12:**
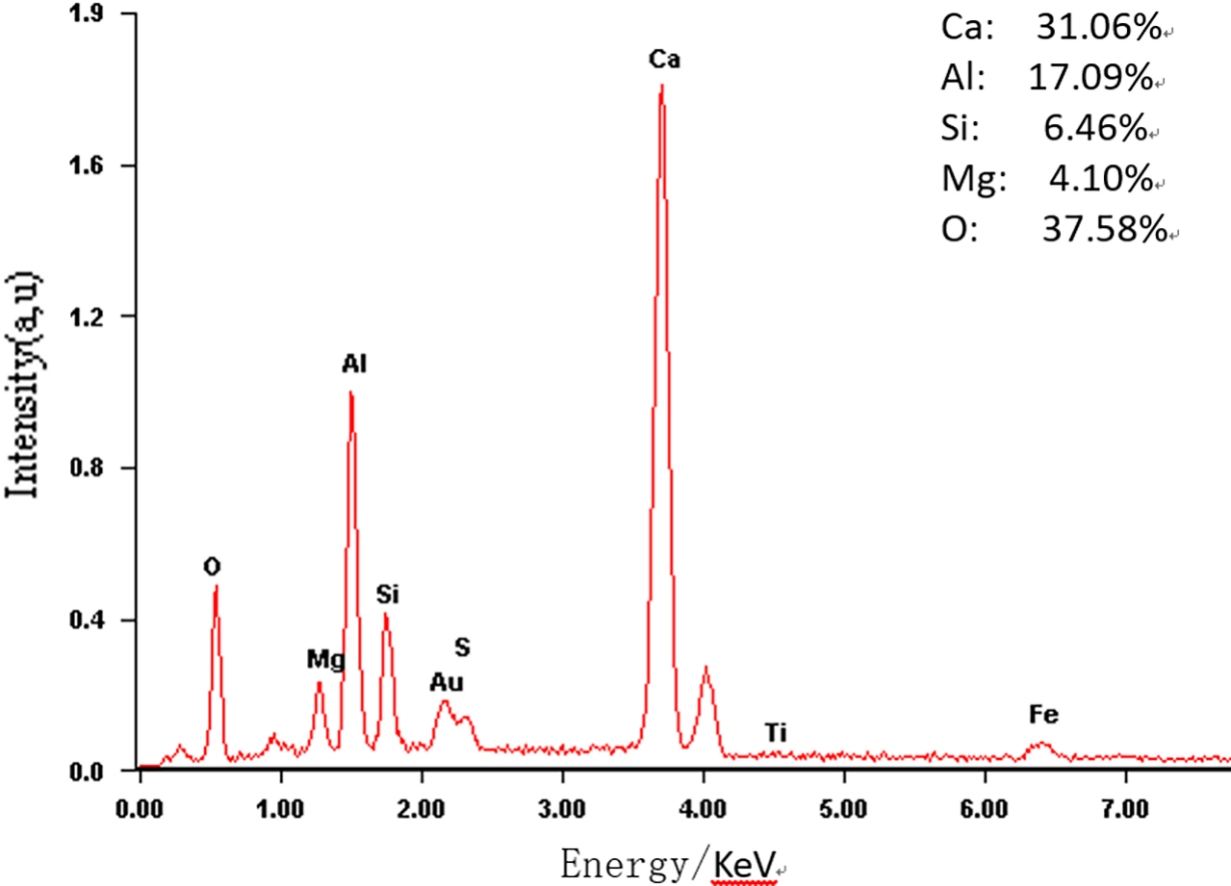
The EDS micrograph of the Q phase in mixed pulverized coal M2.

**Fig 13:**
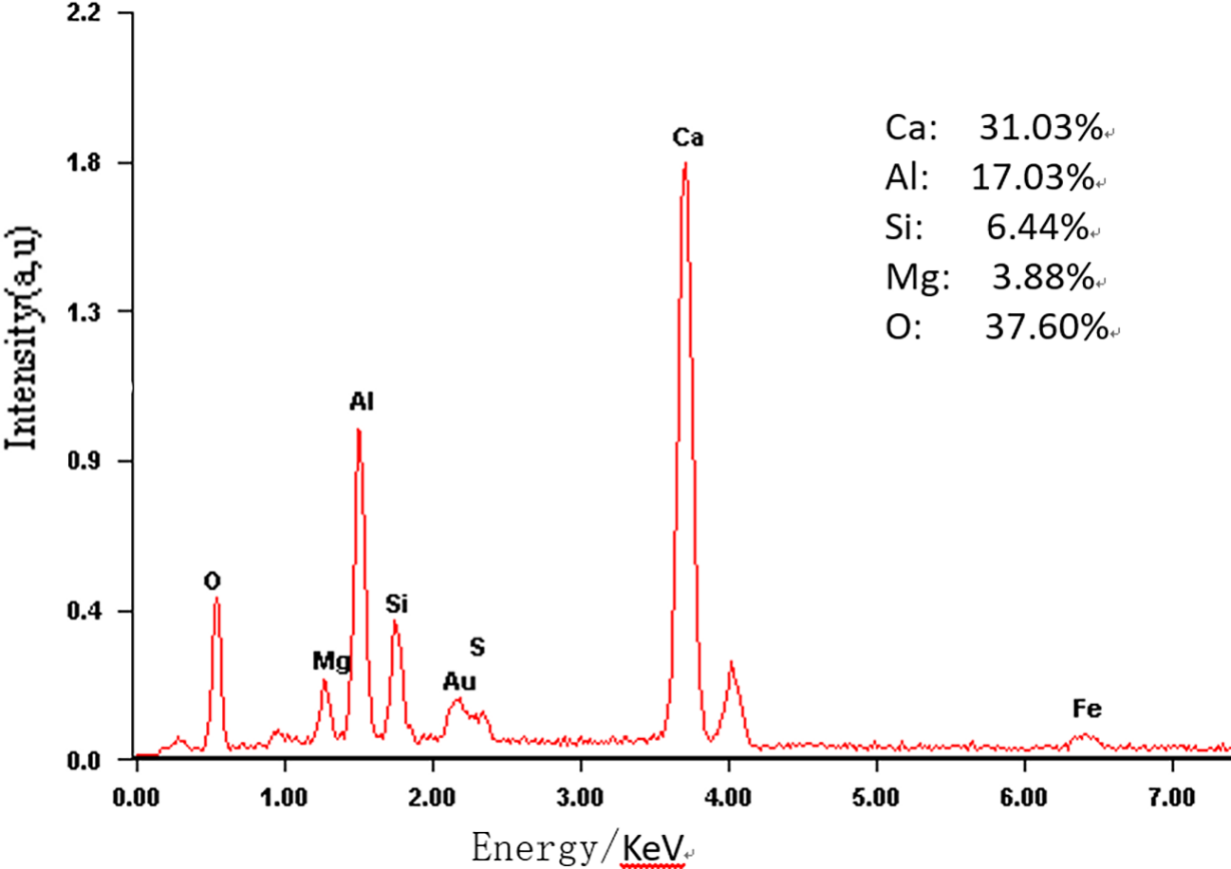
The EDS micrograph of the Q phase in mixed pulverized coal M3.

A comparison of the results in Fig 3, Fig 4, Fig 5, Fig 6 and Fig 7 reveals that the primary minerals in clinker sample M_1_ include 2CaO·SiO_2_, Q phase and 3CaO·3Al_2_O_3_·CaSO_4_. The primary minerals in clinker samples M_2_ and M_3_ are also 2CaO·SiO_2_, Q phase and 3CaO·3Al_2_O_3_·CaSO_4._ However, the proportion of target mineral Q phase in sample M_2_ is higher, and the sample M_3_ contains a higher proportion of 2CaO·SiO_2_. Clinker sample M_4_ and clinker sample M_5_ show slight differences in mineral composition, but the predominant mineral is 2CaO·SiO_2_, while other minerals, such as Q phase and 3CaO·3Al_2_O_3_·CaSO_4_, are present in smaller amounts.

A comparison of Fig 8 and Fig 9 shows that both clinker samples M_6_ and M_7_ contain target minerals 2CaO·SiO_2_ and the mineral Q phase, with high diffraction peaks for CaO.

## 4. Analysis and discussion

### 4.1 Relationship between the mixed coal powder preparation method and mineral production capability

**Fig 14:**
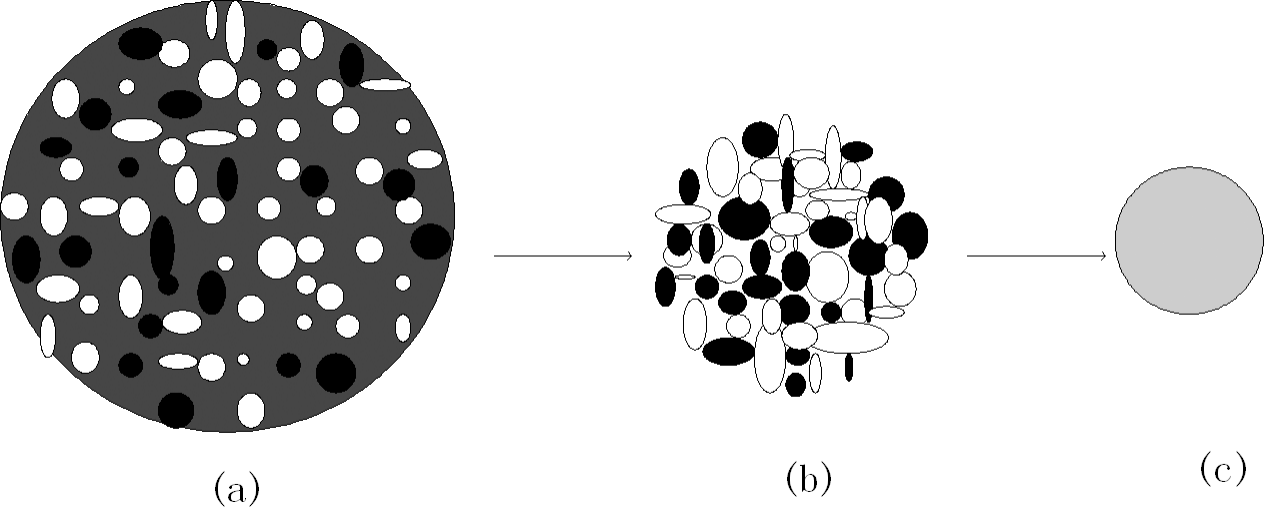
The process diagram for the formation of cement clinker particles from mixed coal powder globular polymerized particles. (a)The moment of the ultra-fast temperature rise of the mixed coal powder globular polymerized particle. (b)Combustible burning and decomposition and aggregation of inorganic mineral. (c)The formation reaction of clinker minerals.

Under these experimental conditions, i.e., the air flow is of 1 m^3^/h, the in-boiler flow rate is of 0.36 m.s^-1^ and the material stays in the boiler for 6.94 s, the clinker mineral formation reactions are relatively complete, and the clinker minerals are well composed. When Particle density *ρ*_p_ = 2000 kg/m^3^, *ρ*_g_ (standard state) = 1.29 kg/m^3^ and the feeding rate reaches 3.5 (g.min^-1^),Solid volume concentration *C*_*v*_ = 0.015%. The particle flow rate in the boiler is assumed to be 0.36 m/s, Then the value of C_v_ will be obtained of0.059%for a sparse suspension. Hence, under these testing conditions, the coal powder multiphase flow in the boiler is a sparse suspension. The mixed coal powder, as a globular polymerized particle, enters the boiler in the form of a dispersed suspension, the heat exchange efficiency improves dramatically, and the temperature rise process completes almost immediately. At the moment of the ultra-fast temperature rise, the mixed coal powder globular polymerized particle is also under the process of volatile matter precipitation and coke combustion. Under the ultra-fast temperature rise and combustion, inorganic minerals in the mixed coal powder globular polymerized particle are experiencing a series of physical changes and chemical reactions in the high-temperature state, including the dehydration and decomposition of coal minerals, the polymerization of newly decomposed and highly active products and additives, such as calcium base, and clinker mineral formation[17].Simultaneously, inorganic minerals in the mixed coal powder globular polymerized particles gradually contract toward the core, and they eventually polymerize into cement clinker particles, with the progress of the clinker mineral formation reaction. Fig 14 shows the process diagram for the formation of cement clinker particles from mixed coal powder globular polymerized particles. In Fig 14(A) and 14(B), light black particles represent combustible substances in the mixed coal powder globular polymerized particles, white particles are calcium base substances, and dark black particles represent coal minerals. In Fig 14(C), gray particles represent clinker minerals.

The test results show that clinker samples M_1_-M_4_ vary significantly in their mineral compositions. As the particle sizes of the coal powder and the additives decrease and the screening level differences between the particle sizes of the coal powder and the additives increase, the proportions of Q phase and 3CaO·3Al_2_O_3_·CaSO_4_ by mass in the clinker increase. Fig 10 and Fig 11 show SEM images for M_2_ and M_3_ mixed coal powders using different preparation methods, respectively. Energy spectrum analysis shows that in these images, the light color represents CaO while the dark color represents the coal. A comparison of Fig 10 and Fig 11 shows that mixed coal powder from the M_2_ preparation method has a more even mixture of CaO and coal. This occurs because, when compared with the M_3_ preparation method, the M_2_ preparation method uses a smaller material particle size, and there are screening level differences between the particle size of the coal powder and the particle sizes of CaO and MgO. The coal and the additives with screening level differences are evenly mixed and grinded, the additives CaO and MgO are more likely to be enclosed by coal powder to form globular polymerized particles, and it also aids in even polymerization and distribution of CaO, MgO and coal minerals, which facilitates the clinker mineral formation reaction of inorganic substances in mixed coal powder. Mixed coal powder particles prepared by reduced particle sizes of coal powder and additives, combined with expanded screening level differences between the coal powder and the additives, provide a physical base for thorough clinker mineral formation reactions.

### 4.2 Relationship between the forms of calcium-based additives and mineral production capability

Compared with the M_5_ clinker sample, the M_6_ clinker sample has higher 2CaO·SiO_2_, mineral Q phase and CaO diffraction peaks, while no diffraction peak of CaCO_3_ is observed. This means that the CaCO_3_ in the mixed coal powder is completely decomposed. However, decomposed CaO does not fully participate in the clinker mineral formation reaction. Hence, the clinker mineral formation reaction is not thorough. This may be caused by different geologic origins of low-calcium limestone and high-calcium limestone, significantly variant lattice structures and different chemical compositions. Variability in these structures and compositions may result in a lower decomposition initialization temperature and decomposition termination temperature for low-calcium limestone than for high-calcium limestone. According to the polymerization reaction theory, the lower decomposition point of low-calcium limestone results in easier decomposition of highly active CaO from low-calcium limestone, which instantly initiates a clinker mineral formation reaction with oxide decomposed from the coal mineral. Because a mix of CaCO_3_ will lead to an incomplete clinker mineral formation reaction and will significantly lower the proportion of coal powder in the mixed coal powder, and the heat value of the mixed coal powder may be significantly degraded. Under the present conditions, it is not the optimum choice to substitute CaCO_3_ for CaO in the Q phase cement clinker coproduction. Before the technology becomes mature and gains practical application, further research on the forms of calcium-based additives and mixing processes for the cement clinker coproduction is required. The generation equation of Q phase as follows.

$$2CaO\cdot Al_{2}O_{3}\cdot SiO_{2}+CaO\to 2CaO\cdot SiO_{2}+CaO\cdot Al_{2}O_{3}$$ (1)

$$12CaO\cdot 7Al_{2}O_{3}+5Al_{2}O_{3}\to 12(CaO\cdot Al_{2}O_{3})$$ (2)

$$4(CaO\cdot Al_{2}O_{3})+2CaO_{2}\cdot SiO_{2}+MgO\to 6CaO\cdot 4Al_{2}O_{3}\cdot MgO\cdot SiO_{2}$$ (3)

## 5. Conclusions

The experimental study on the relationship between the mineral production capability and the physiochemical properties are completed in a two-stage multiphase reaction test bed with Changguang coal, and the main conclusions are obtained as follows:

1. As the particle sizes of the coal powder and the additives decrease and the screening level differences between the particle sizes of the coal powder and the additives increase, the proportion of Q phase and 3CaO·3Al_2_O_3_·CaSO_4_ by mass in the clinker increases. When the methods of reducing particle sizes of the coal powder and the additives and expanding screening level differences between the coal powder and the additives are used to prepare mixed coal powder particles, the additives of CaO and MgO are more likely to be enclosed by coal powder to form globular polymerized particles. This encapsulation also aids the polymerization and even distribution of CaO, MgO and coal minerals, which facilitates the clinker mineral formation reaction of inorganic substances in the mixed coal powder.
2. Target minerals, such as 2CaO·SiO2 and Q phase, are found in both industrial high-calcium limestone and low-calcium limestone coproduced clinker samples. A diffraction peak of free CaO is also evident in both samples. Compared with a coproduced clinker sample of high-calcium limestone, that of low-calcium limestone exhibits higher diffraction peaks for 2CaO·SiO2 and Q phase.
3. With the current state of the art, it is not yet the optimum choice to substitute CaCO3 for CaO in Q-phase cement clinker coproduction. Before the technology matures and gains practical application, further study on the form and the mixing process of calcium-based additives for cement clinker coproduction is necessary.
